# Broadband EPR Spectroscopy of the Triplet State: Multi-Frequency Analysis of Copper Acetate Monohydrate

**DOI:** 10.3390/ijms241914793

**Published:** 2023-09-30

**Authors:** Wilfred R. Hagen

**Affiliations:** Department of Biotechnology, Delft University of Technology, Building 58, Van der Maasweg 9, 2629 HZ Delft, The Netherlands; w.r.hagen@tudelft.nl

**Keywords:** broadband EPR, triplet state, copper acetate monohydrate, dinuclear copper complex, inverted Orbach relaxation, Raman relaxation, zero-field parameter distribution, dipolar interaction, zero-field transition, double-quantum transition

## Abstract

Electron paramagnetic resonance spectroscopy is a long-standing method for the exploration of electronic structures of transition ion complexes. The difficulty of its analysis varies considerably, not only with the nature of the spin system, but more so with the relative magnitudes of the magnetic interactions to which the spin is subject, where particularly challenging cases ensue when two interactions are of comparable magnitude. A case in point is the triplet system S = 1 of coordination complexes with two unpaired electrons when the electronic Zeeman interaction and the electronic zero-field interaction are similar in strength. This situation occurs in the X-band spectra of the thermally excited triplet state of dinuclear copper(II) complexes, exemplified by copper acetate monohydrate. In this study, applicability of the recently developed low-frequency broadband EPR spectrometer to S = 1 systems is investigated on the analysis of multi-frequency, 0.5–16 GHz, data from [Cu(CH_3_COO)_2_H_2_O]_2_. Global fitting affords the spin Hamiltonian parameters *g_z_* = 2.365 ± 0.008; *g_y_* = 2.055 ± 0.010; *g_x_* = 2.077 ± 0.005; *A_z_* = 64 gauss; *D* = 0.335 ± 0.002 cm^−1^; *E* = 0.0105 ± 0.0003 cm^−1^. The latter two define zero-field absorptions at ca. 630, 7730, and 10,360 MHz, which show up in the spectra as one half of a sharpened symmetrical line. Overall, the EPR line shape is Lorentzian, reflecting spin-lattice relaxation, which is a combination of an unusual, essentially temperature-independent, inverted Orbach process via the S = 0 ground state, and a Raman process proportional to T^2^. Other broadening mechanisms are limited to at best minor contributions from a distribution in E values, and from dipolar interaction with neighboring copper pairs. Monitoring of a first-order double-quantum transition between 8 and 35 GHz shows a previously unnoticed very complex line shape behavior, which should be the subject of future research.

## 1. Introduction

Electron paramagnetic resonance spectrometers commonly employ magnetic-field, *B*, scanning in combination with a cavity resonating at a single, fixed microwave frequency, *ν*, usually in the X-band at ca 9.5 GHz [1]. In multi-frequency EPR typically two or more single-frequency instruments are used in combination, covering a few octaves of frequency range [2,3], with the goal of collecting data sets that would allow for unequivocal interpretation of molecular paramagnetism. The underlying concept is that all but the simplest spin systems are subject to a combination of magnetic interactions, some of which are linear in the frequency and the field, while others are independent of the frequency [4]. In high-frequency EPR (*ν* circa ≥ 100 GHz) this may be implemented by switching between several microwave sources to step through a frequency range of over two octaves [5,6,7,8]. For more conventional frequencies, around the standard X-band, I have developed a broadband EPR spectrometer [9,10,11,12,13,14] as a convenient, affordable single station, using a conventional electromagnet, whose resonator circuit can be tuned to many frequencies over the approximate range of 0.1 to 18 GHz, that is, over some seven to eight octaves. Here, I describe application of the broadband methodology to the analysis of triplet-state EPR.

A triplet-state molecule is a system with two unpaired electrons forming a total spin of *S* = 1, and with substates |*m*_S_ = 1>, |*m*_S_ = 0>, |*m*_S_ = −1>, which is minimally characterized by the spin Hamiltonian:

(1) H=S·D·S+βB·g·S

**Figure 1:**
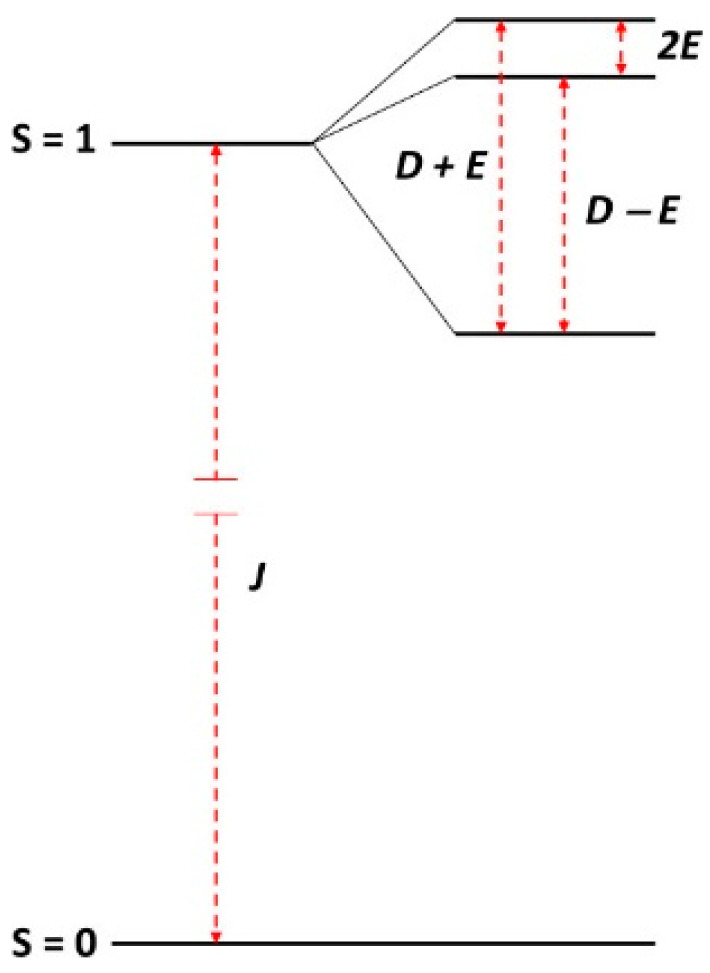
Zero-field levels of the triplet system. Depending on the molecular system, the frequencies of the three zero-field transitions within the S = 1 manifold can range from sub-microwave (2*E* < 300 MHz) [17] to super-microwave (*D* + *E* > 300 GHz) [18]. The singlet to triplet transition is not observed, as it typically lies at a much higher frequency in the far infrared, and it is spin-forbidden.

The first term describes the electron spin–spin interaction, which is independent of *ν*, and the second term describes the electronic Zeeman interaction, which is linear in *ν*. *S* is a spin operator, ***D*** is the 3 × 3 zero-field matrix, *β* is the Bohr magneton, and ***g*** is the 3 × 3 matrix of g values. Additional terms in the Hamiltonian may be required to describe, e.g., statistical distributions in the elements of ***D*** and ***g***, hyperfine interactions, and intermolecular dipolar interactions (below). In the principal axes system in which ***D*** is diagonal, its trace may be set equal to zero [15], which implies that there are only two independent tensor elements, and so the interaction may also be written as:

(2) $$H=D\left[S_{z}^{2}-S(S+1\right)/3]+E(S_{x}^{2}-S_{y}^{2})$$

wherein *D* and *E* are the axial and rhombic zero-field splitting parameters, and 0 ≤ *E* ≤ *D*/3 [16]. In the absence of an external magnetic field the spin–spin interaction gives rise to a resolution of the otherwise degenerate triplet spin levels, affording zero-field splittings equal to *D* + *E*, *D* − *E*, and 2*E* (Figure 1). An implication of this level scheme for broadband EPR is that there should be three microwave frequencies for which a transition occurs exactly at zero field.

**Figure 2:**
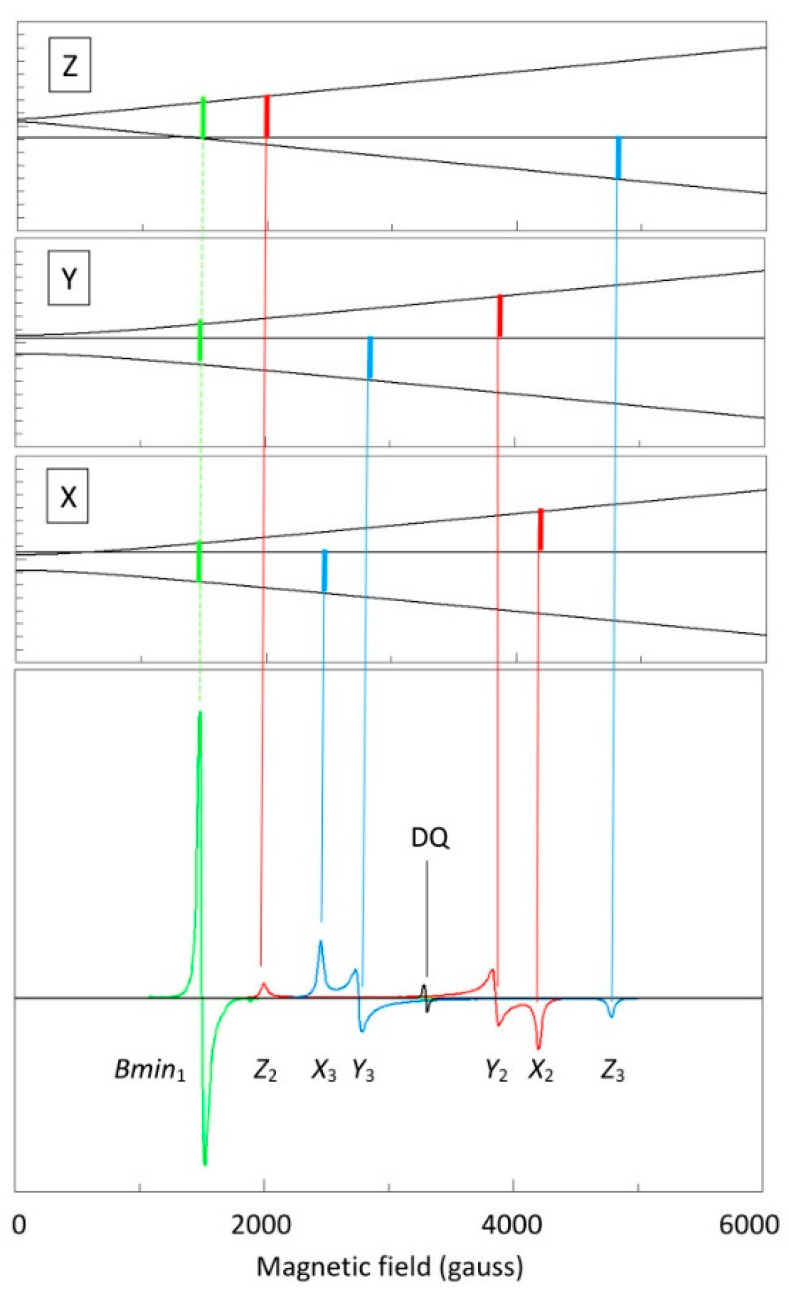
Labelling of the triplet EPR transitions. An example perpendicular-mode X-band, 9.5 GHz, EPR spectrum, with *g_iso_* = 2.00, *D* = 0.13 cm^−1^ and *E* = −0.01 cm^−1^ is used to illustrate the labelling of the four possible transitions according to Kottis and Lefebre [19,20] in the order in which they occur in a positive magnetic-field scan. X, Y, Z are the canonical orientations of the zero-field tensor. The first transition (green) is the Δ*m_S_* = 2, or half-field transition, which for the given parameters is forbidden along the axes, but has an off-axis turning point at *B_min_*. The second (red) and third (blue) are the regular Δ*m_S_* = 1 transitions along the axes. Changing the sign of one of the parameters *D*, *E*, and exchanging the values of *g_x_* and *g_y_*, as well as the values of *W_x_* and *W_y_*, will interchange the labels X_I_ and Y_i_. The fourth (black) is the double-quantum transition that occurs at intermediate orientations when the three energy levels happen to be separated by equidistant energies.

When the *B*-field is turned on and scanned, this fine structure defines three regimes of triplet spectra, namely, for *D* < *hν*, *D* ≈ *hν*, and *D* > *hν*. When the first inequality holds, *D* < *hν*, then all three transitions, |−1> → |+1>, |0> → |+1>, and |−1> → |0> are readily observable. This is illustrated in Figure 2 with a calculated X-band (*hν* ≈ 0.32 cm^−1^) spectrum for *g_x_* = *g_y_* = *g_z_* = 2, *D* = 0.13 cm^−1^, and *E* = 0.01 cm^−1^. Consistency of notation in the labelling of transitions is of the essence in the interpretation of multi-frequency EPR of the triplet state. For example, in a high-frequency study on an S = 1 system Krzystek et al. claim to use “standard nomenclature for triplet states”, for which they reference papers by Wasserman et al. [17] and by Kottis and Lefebre [19]. However, the two papers describe two different mutually exclusive labelling systems, and Krzystek et al. use a third system different from the two cited ones [18]. I follow the notation of Kottis and Lefebre [19,20], who number transitions according to their first appearance when increasing the magnetic field from zero onwards in a *D* < *hν* spectrum (cf. Figure 2). Note that changing the sign in either one of the zero-field parameters, *D* and *E*, will interchange the labels X and Y for the turning points in transitions 2 (|0> → |+1>) and 3 (|−1> → |0>).

The |−1> → |+1> transition 1 is sometimes called the ‘half-field’ transition as it is found at a resonance field corresponding to half the field value of the center of gravity of the transitions 2 and 3 when *D* << *hν* (which corresponds very approximately to the field value for 2× *g*). It is also called the ‘forbidden’ transition as it formally involves a ∆*m_S_* = 2 quantum change in high-field notation. It may be singled out by observation in parallel mode, that is with the microwave vector oriented parallel to the external field vector [21]. For the present work it is of importance to note that for the spin Hamiltonian parameters and X-band frequency of Figure 2, transition 1 has zero intensity (transition probability) in a regular, normal-mode resonator, for which the microwave magnetic component is perpendicular to the external magnetic field (*B*_1_ ⊥ *B*), in all of the canonical directions X, Y, Z; however, there is an extra turning point at *B_min_*, which is a minimum field value below the resonant fields for the X, Y, Z, directions, which occurs at intermediate orientations, and at which the spectrum does have finite transition probability. This is the reason why the low-field feature in Figure 2 is labelled *B_min_*_1_, and the labels *X*_1_, *Y*_1_, *Z*_1_ are absent.

A fourth, so-called double-quantum, transition is also frequently detected (cf. Figure 2): it occurs when ∆*E*(|−1>,|0>) is exactly equal to ∆*E*(|0>,|1>), which holds only for specific intermediate orientations (cf. [19,20,22]).

In the X-band, the *D* < *hν* case is exemplified by many aromatic phosphorescent biradicals produced by continuous near-UV irradiation [17,23].

Contrarily, when *D* > *hν*, then at least some transitions along the principal axes, and also the double-quantum transition, are not possible because the energy separation between spin levels exceeds the energy of the microwave, and all observable transitions may well occur at inaccessibly high magnetic fields. In the latter situation, the triplet system is called EPR silent. The *D* > *hν* case in the X-band is represented by the class of mononuclear high-spin Ni(II) compounds [22,24]. Although these complexes are usually studied in the form of pure compounds, which implies a very high paramagnet concentration (molar order of magnitude), their X-band spectra, if any, are typically of limited intensity.

The intermediate *D* ≈ *hν* condition results in asymmetric spectra in which the double-quantum transition has relatively reduced intensity, in which some transitions between two spin levels may occur twice, that is at two different field values, and in which characteristic features are observed near zero magnetic field. The latter may be singled out and amplified by observation in parallel mode. *D* ≈ *hν* in the X-band holds for the class of exchange-coupled dimers of Cu^2+^ ions [25,26,27]. This intermediate case is particularly interesting for broadband EPR, in which it can be either converted to a *D* < *hν* case by an increase in the microwave frequency, or to a *D* < *hν* case by a reduction in the frequency. For these systems, the broadband spectrometer can generate sets of spectra that widely vary in shape, thus providing a rich data set whose analysis can be unequivocal and accurate in terms of spin Hamiltonian parameters. Here, as an illustration of the methodology, I scrutinize solid microcrystalline copper(II) acetate monohydrate dimer, Cu_2_(CH_3_COO)_4_·2H_2_O, as an example of a *D* ≈ *hν* at X-band.

## 2. Results and Discussion

### 2.1. Description of the Model

Copper acetate is a ‘classic’ in the history of EPR spectroscopy as it was the first compound for which electron exchange between metal centers was experimentally observed in the form of an excited-state system spin S = 1 resulting from parallel coupling of two S = 1/2 spins from individual Cu^2+^ ions, and with a hyperfine pattern of seven lines in 1:2:3:4:3:2:1 intensity pattern as a consequence of electron delocalization over two copper nuclei with individual nuclear spin I = 3/2 [25,28,29,30]. Susceptibility measurements showed the favored coupling to be anti-parallel, resulting in a diamagnetic ground state with S = 0 separated by some 292 cm^−1^ from the parallel-coupled S = 1 excited state [25,31]. Shortly afterwards, its crystal structure was determined, with a short Cu-Cu distance of 2.61 Å [31,32,33,34]. Whether this distance represents a real chemical bond has remained under debate [35,36,37].

**Figure 3:**
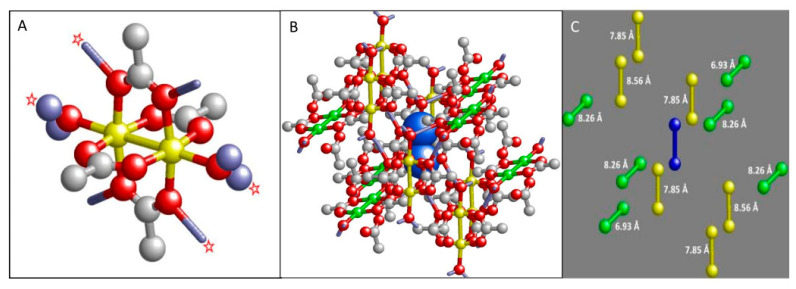
Structure of copper acetate monohydrate. (**A**) The monomer unit has quasi-axial symmetry with four perpendicular acetate bridges and two terminal waters. Rhombicity is induced by the fact that only two of the four bridges form hydrogen bonds with the water ligands from neighboring units. Additional asymmetry comes from the different lengths of the H-bonds, where the four starred ones are 1.86 Å, and the other four are 2.00 Å. Methyl hydrogens do not participate in hydrogen bonding, and are left out. (**B**) A copper pair (blue) is surrounded by 12 nearest neighbors in a pattern of two (yellow and green) interwoven fabrics. The center-of-gravity distances are expected to show up as dipolar interaction in EPR spectra. The figure is constructed from crystallographic data in [33] as detailed in Materials and Methods Section 3. (**C**) The structure in (**B**) simplified to copper pairs only, with center-of-gravity distances to the central blue pair indicated.

The structure of a single copper acetate molecule is given in Figure 3A. The two Cu^2+^ ions are bridged by four acetato ligands affording an axially symmetric structure with the symmetry axis (z-axis) defined by the copper atoms and the oxygen atoms of the end-on water ligands. The water hydrogen atoms form hydrogen bridges (1.9 −2.0 Å), indicated by pale-blue sticks, with acetate β oxygens of neighboring molecules; equally, acetate oxygens from the presented molecule form bridges with water molecules from neighboring molecules. Since these bridges are only formed by two (mirror images) out of four acetates, a deformation is introduced in the structure, which should be detectable as *g_x_* ≠ *g_y_* and *E* ≠ 0 in the EPR.

The molecule is surrounded by two types of neighbors as illustrated in Figure 3B,C. One half of the neighbors (yellow copper pairs) has their molecular axis system co-linear with that of the central molecule (blue copper pair). The other half (green copper pairs) have their axes rotated with respect to the central one. The figure depicts a total of 12 neighbors because these all have their center of gravity at a distance of less than 10 Å from that of the central molecule; therefore, they can be expected to contribute significantly to the linewidth of the EPR spectra via intermolecular dipolar interaction.

### 2.2. Temperature Dependence of Copper Acetate X-Band EPR

**Figure 4:**
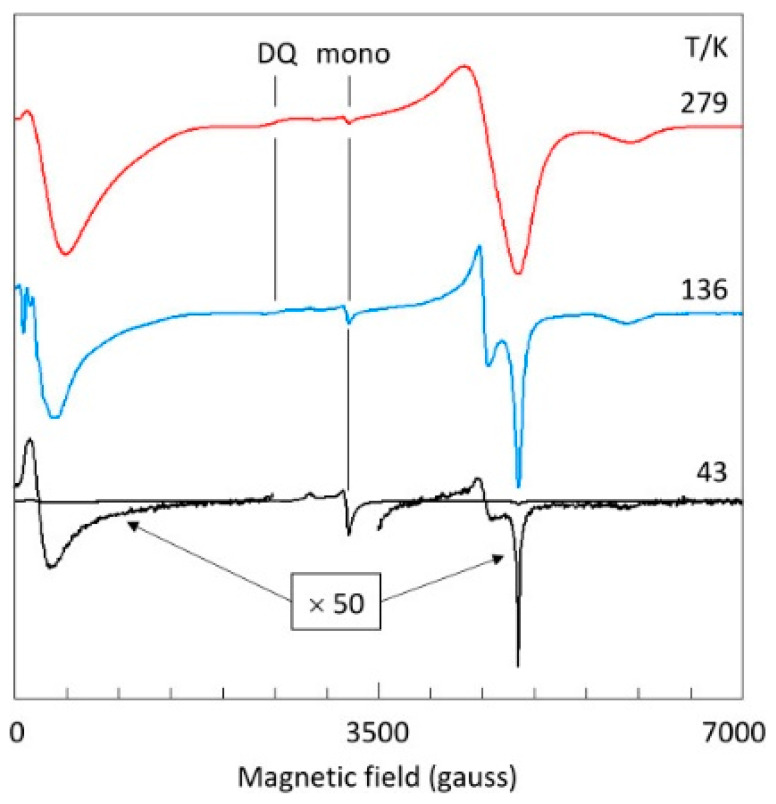
Temperature dependence of the X-band EPR of copper acetate. The spectra illustrate several points of interest. The signal-to-noise ratio rapidly decreases with decreasing temperature due to depopulation of the excited-state triplet. Concomitantly, the spectral resolution increases, but at the lowest temperature resolution is again reduced for some features of the spectrum, notably near zero field and around 4.5 kgauss. The amplitude of a signal from monomeric copper sites increases with decreasing temperature, following Curie’s law. A signal assigned to a double-quantum transition has apparent negative amplitude. It is most pronounced at ambient temperature, where broad lines afford significant overlap of the two Δ*m_S_* = 1 transitions for certain intermediate orientations of the magnetic-field vector in the axis system of the zero-field interaction. EPR conditions: microwave frequency, 9414 MHz; microwave power, −10 dB of 200 mW; modulation frequency, 100 kHz; modulation amplitude, 1 gauss.

As a first orientation to the spectroscopy, Figure 4 shows the X-band spectrum at three temperatures. A pronounced reduction in linewidth is seen with decreasing temperature, which could come from attenuation of dipole–dipole interaction caused by reduction in paramagnetic neighbor concentration due to depopulation of the triplet state and/or from lifetime broadening at higher temperatures. The figure indicates that at high spectral resolution the intensity drops to a very low level, which would be undesirable for broadband studies. Mononuclear copper contaminant (‘mono’) is also indicated in Figure 4. Its origin is not known; it is presumably associated with surface positions of the microcrystals. As an S = 1/2 system, its intensity increases with decreasing temperature: from a very minor feature at room temperature, it has become the dominant spectral component at 43 K. A double-quantum (DQ) transition is observed at higher temperatures. Its unusual negative-absorption spectral shape will be addressed in Section 2.6.

**Figure 5:**
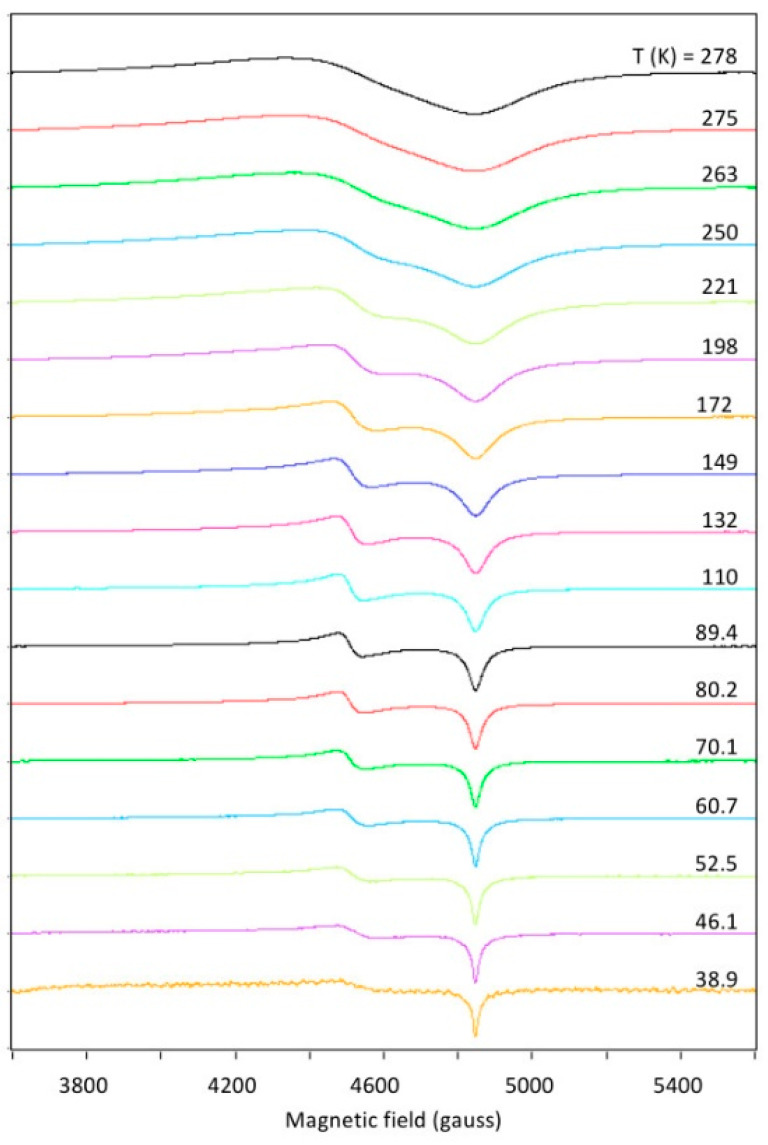
Details of temperature variation of the *Y*_2_*X*_2_ features. In a gradual decrease in sample temperature from 278 to 43 K, the *X*_2_ transition at 4847 gauss is seen to sharpen in a smooth manner, while, contrarily, the *Y*_2_ transition at 4510 gauss sharpens down to an intermediate temperature of circa 150 K, and then starts to broaden again. EPR conditions as in Figure 4. All signals are non-saturating. Amplitudes are normalized to unity.

**Figure 6:**
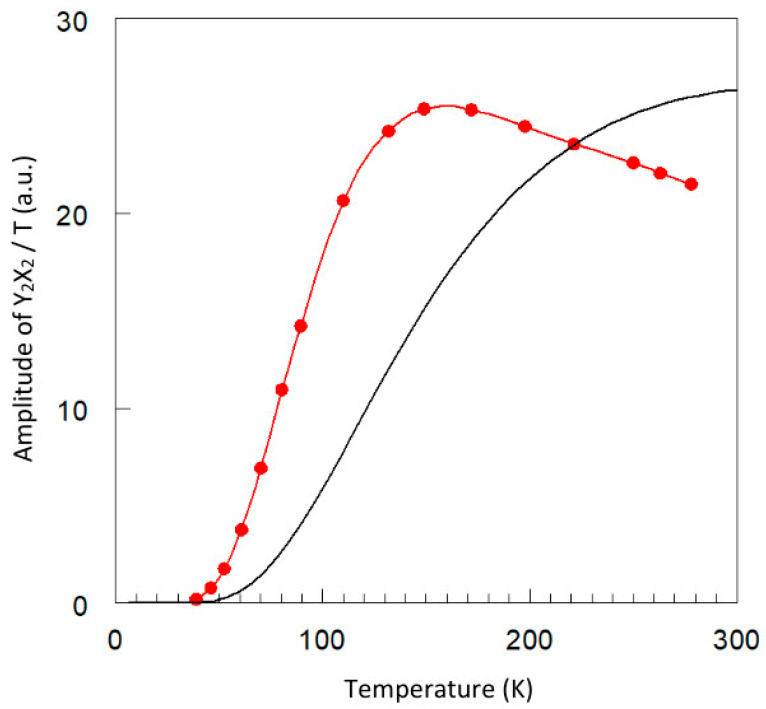
Comparison of triplet population level and normalized EPR intensity. The black line is the triplet population calculated with Equation (3). The red dots are real amplitudes of the spectra in Figure 5 divided by the absolute temperature. The red curve is a spline fit to the data. Its maximum is adjusted to that of the population curve. The shapes of the two curves differ because the red one is the result of depopulation combined with line sharpening with decreasing temperature. Its maximum, around 150 K, is taken as a compromise temperature for a broadband EPR study, because both signal-to-noise ratio and spectral resolution at this temperature are acceptable, and broadening of the *Y*_2_ feature and the specific line shape of the *X*_2_ feature at temperatures below 150 K are not understood (see also Figure 7).

Spectral resolution is considered in more detail in Figure 5 which monitors the shape of the *Y*_2_- and *X*_2_-feature of the |0> → |1> transition-2 as a function of temperature. The fractional population of the triplet state in a Boltzmann distribution over the S = 0 and S = 1 states is:

(3) $$N_{triplet}=\frac{1}{\left(1/3\right){exp}^{+∆/kT}+1}$$

in which ∆ ≈ 292 cm^−1^ is the singlet-to-triplet energy splitting from exchange interaction. The peak-to-peak amplitude of the overall *YX* feature plotted in Figure 6 can be seen to be quite different from the triplet depopulation curve because it is the result of a combination of depopulation and line sharpening. A maximum in absolute intensity is found around 150 K, and with a concomitant reasonable spectral resolution (Figure 5) this temperature is taken to be practical for broadband measurements.

**Figure 7:**
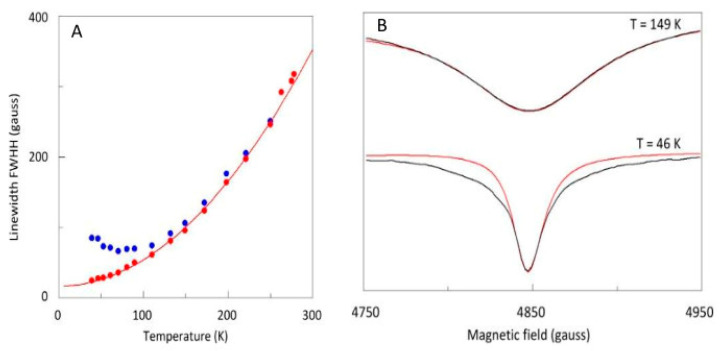
Linewidths of the *Y*_2_ and *X*_2_ features as a function of temperature. (**A**) For the *X*_2_ transition the linewidth (red dots) is measured as full width at half height. The width (blue dots) of the *Y*_2_ transition is obtained from the amplitude ratio of the two transitions with a calibration correction determined by simulation of the spectrum at 149 K using a Lorentzian line shape. The red line is a fit to the *X*_2_ data according to Equation (4), which assumes the linewidth to reflect spin-lattice relaxation via a virtually temperature-independent, inverse (that is, to the S = 0 ground state) Orbach mechanism, and a T^2^-dependent Raman mechanism. The *Y*_2_ linewidth can be seen to re-broaden at the lowest temperatures. (**B**) Simulation shows that the *X*_2_ peak is a perfect Lorentzian at (and above) 149 K; however. it exhibits an unknown line shape at the lowest temperatures, whose wings fall off less rapidly than those of a Lorentzian. EPR conditions as in Figure 4.

**Figure 8:**
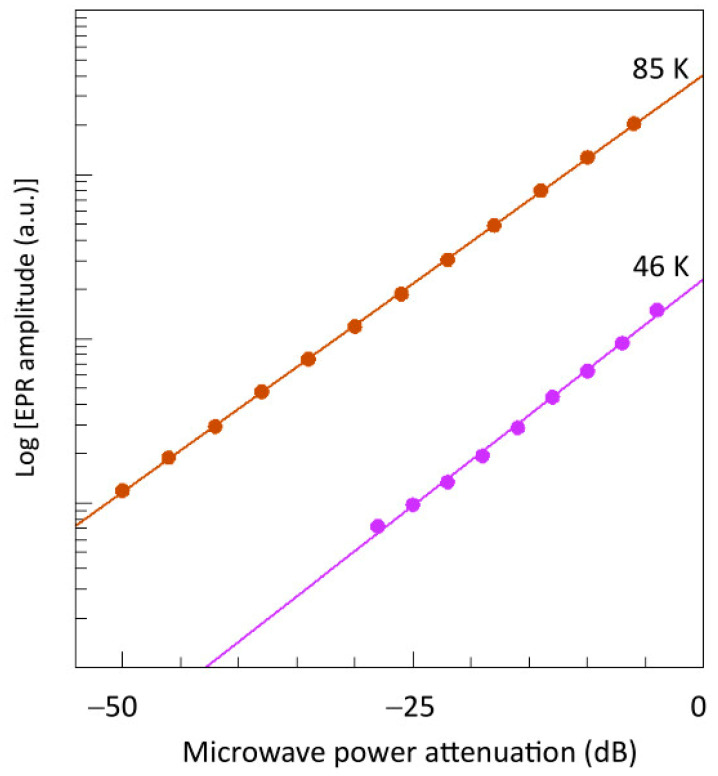
Power plots of the *Y*_2_*X*_2_ feature. The logarithm of the amplitude of the *Y*_2_*X*_x_ feature at 85 K (brown) and 46 K (violet) is plotted versus the microwave power in dB attenuation from 200 mW. Linearity of the data shows that the signal does not saturate up to full power at the two temperatures.

There is another reason to exclude the low-temperature data of apparently high resolution from analysis. In Figure 7, I plot the linewidth (FWHH) of the *Y*_2_ and *X*_2_ feature as a function of temperature. The *X*_2_ negative peak becomes monotonically sharper with decreasing temperature; however, sharpening of the *Y*_2_ derivative-shaped peak levels off approximately below 110 K, and below 70 K even increases with decreasing temperature. In other words, in this part of the spectrum the resolution in fact decreases with decreasing temperature. This effect is also seen in the near-zero-field line in Figure 4. The phenomenon is unrelated to saturation since both *Y*_1_ and *X*_1_ features do not saturate: their shapes are unaffected by increasing microwave power levels and their amplitudes are linear in the power in –dB units (Figure 8). Furthermore, as can be seen in Figure 7B, the shape of the *X*_1_ feature closely resembles a Lorentzian line shape at 149 K; however, at 46 K the experimental shape falls off less rapidly in the wings than a Lorentzian would. At this temperature, dipolar broadening is negligible (see below).

Spin-lattice relaxation, or T_1_ relaxation, in ionic crystals is typically described by a combination of direct, Orbach, and Raman processes, which involve, respectively, coupling to a single phonon, to two phonons, or the inelastic scattering of a phonon [15]. Direct processes are limited to low temperatures, T < 10 K and are thus unobservable in the present case. The Orbach mechanism was originally proposed for relaxation via an excited state whose energy, *∆*, is less than the maximum phonon energy [38], but it was later proposed, and experimentally verified, that the intermediate state may also be lower in energy than the magnetic levels of the EPR transition, such as a singlet ground state in a singlet/triplet system [39,40]. In contrast to the strongly temperature-dependent process originally proposed by Orbach, the inverse-Orbach relaxation rate is nearly independent of the temperature. For non-Kramers systems, the Raman process affords a T^7^ dependence at low temperatures, which changes to a T^2^ dependence in the high-temperature limit [15]. Linewidth data from the X_2_ peak in Figure 7 can be fitted to the equation:

(4) $$\frac{1}{T_{1}}=\frac{R_{0}}{1-\exp(-\frac{J}{kT})}+R_{R}T^{2}$$

which suggests a combination of an inverted Orbach and a Raman relaxation under the assumption that the linewidth has no contribution from inhomogeneous broadening mechanisms. This assumption remains to be checked, below.

### 2.3. Broadband EPR of Copper Acetate

**Figure 9:**
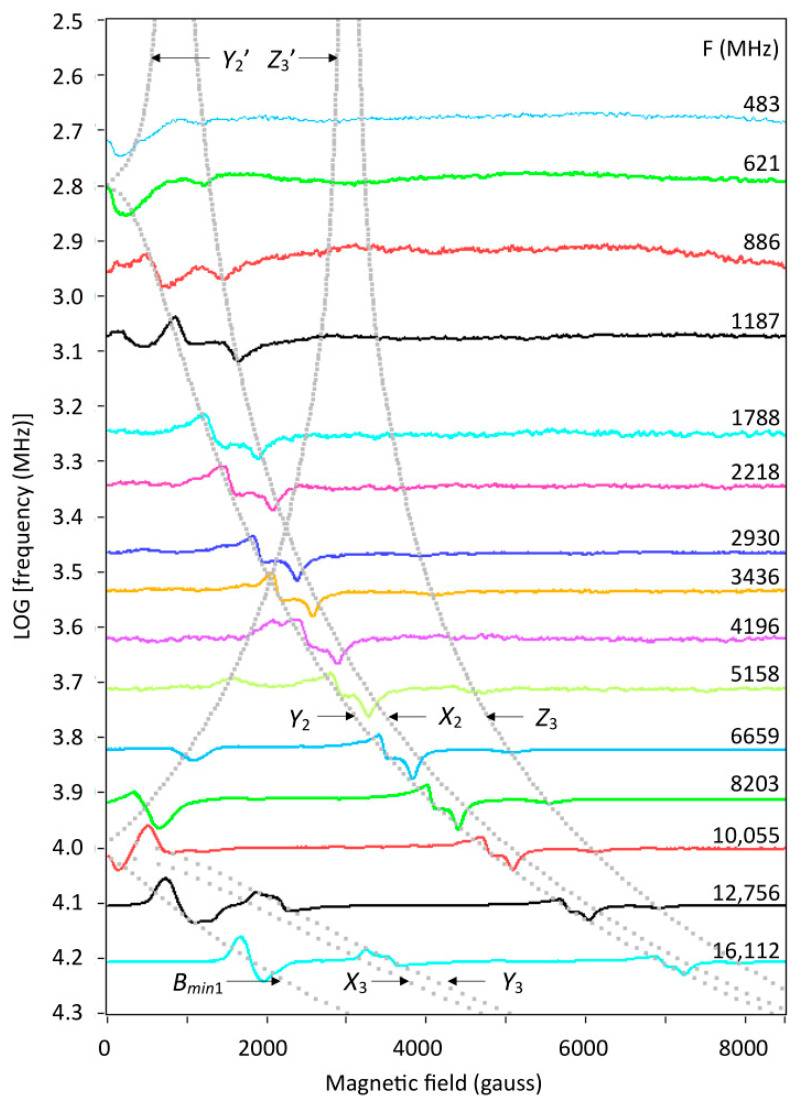
Overview of copper acetate monohydrate broadband EPR spectroscopy. Spectra have been taken at frequencies between 483 and 16,112 MHz and have been plotted on a linear field scale. The baseline of each spectrum has been shifted to the logarithm of the frequency. The dotted grey lines are canonical resonance positions calculated with the program CanonicalTripletResonances with omission of all features with amplitude (transition probability) equal to zero. Their labels correspond to the labels defined in the triplet example spectrum in Figure 2. Primed symbols are for ‘second resonances’ that occur at some frequencies due to energy-level crossing as a function of magnetic field. At high frequencies a dotted line for Z_1_ is omitted, because the feature is overwhelmed by the B_min1_ feature. EPR conditions: microwave frequency as indicated; incident microwave power, 9–15 dBm; reflected power at the detection diode, 3–8.5 dBm; modulation frequency, 100 kHz; modulation amplitude, 25 gauss; temperature, 150 K.

An overview of broadband EPR data taken at 150 K is given in Figure 9, which reveals several features of interest. The stack of spectra is not normalized on a reciprocal *g*-scale because none of the spectral features correspond to real g-values. For the spacing of spectra, a logarithmic frequency scale is used. The high-field pattern of xy features from the |0> → |1> transition-2 and the weak, negative *g_z_* peak from the |−1> → |0> transition-3 can be seen to spread out over an increasing field range with decreasing frequency. This continuous change in effective g-values should be a good test system for the determination of spin Hamiltonian parameters by global fitting of broadband data. Matters are more complex at low field, where both the half-field transition-1 and the *XY* lines of the transition-3 move with decreasing frequency into zero field ca. at X-band frequencies. These zero crossings correspond to energies *D* ± *E*, and so the value of |*D*| is estimated to be circa 10 GHz or some 0.34 cm^−1^. Upon further frequency decrease, one transition bounces back from zero field and moves towards higher field. At very low frequencies also one of the 2-transitions moves into zero field at circa 0.6 GHz, therefore |2*E*| ≈ 0.02 cm^−1^. The complete set of spectra in Figure 8 represents transition from the case *D* < *hν* through *D* ≈ *hν* into the *D* > *hν* case.

The dotted gray lines trace the triplet transitions along the canonical orientations, that is, the x, y, and z axes of the colinear Hamiltonian in Equation (1) plus the *B_min_*_1_ orientation, all illustrated in Figure 2. The lines are based on a parameter set determined by simulation of individual spectra (see below). The lines are labelled as in Figure 2. Not all possible transitions are represented. The transitions *X*_1_, *Y*_1_, and *Z*_2_ have zero transition probability for the specific spin Hamiltonian parameter set with the microwave perpendicular to the external magnetic field. The *Z*_1_ transition is omitted because it is close to the *B_min_*_1_ transition, and is completely overshadowed by the latter. The labels *Y*_2′_ and *Z*_3′_ are primed to indicate that for certain field-frequency combinations a transition can occur twice due to crossing of energy levels. A similar triplet analysis has been made by Krzystek et al. at high frequencies on a V(III) complex with very large zero-field splittings. Technically, that study differs from the present one in that the sample was in an oversized-pipe wave propagation system, in which the orientation of the microwave *B*_1_ field with respect to the external magnetic field B was poorly defined, and thus transitions that would have been strictly forbidden in perpendicular-mode resonators (as used in the present study) gained some finite intensity [18].

### 2.4. Spectral Simulation

**Figure 10:**
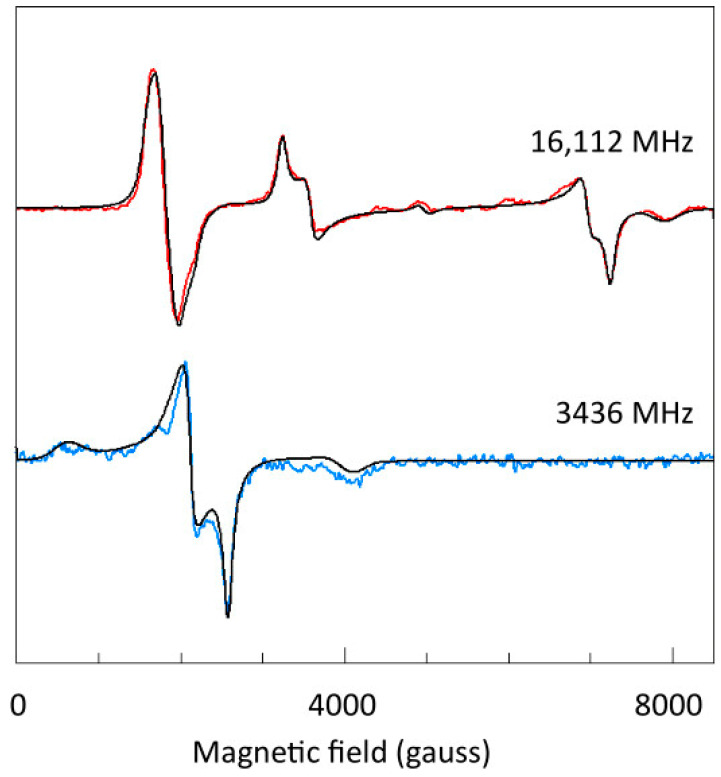
Simulation of triplet EPR spectra taken at two widely different frequencies. Simulation of the spectrum at 16,122 MHz with the program BB-TRIPLET was optimized by visual inspection, affording the parameters given in Table 1. Those parameters were then used without further optimization to generate a simulation of the spectrum at 3436 MHz. The fit indicates that the linewidths in x-, y-, and z-directions are fully independent of the microwave frequency. Experimental spectra were taken from Figure 9.

Figure 10, upper trace, gives an optimized simulation of the 16.11 GHz spectrum, based on the parameters given in Table 1.

A weak double-quantum transition is found in the spectrum and in its simulation (see Section 2.6, below). The *B_min_*_1_ and *Z*_3_ features are broadened by hyperfine splitting from the two copper nuclei, whose value will be verified in Section 2.7. The program BB-TRIPLET is explained in Section 3, Materials and Methods, and listed in Supplementary Information. The simulation is based on a Lorentzian line shape, from lifetime broadening, with slight linewidth anisotropy. In the lower trace of Figure 9, it can be seen that exactly the same parameter set also affords a good fit to the 3.44 GHz spectrum. We can, in consequence, conclude that any broadening mechanism linear in the frequency, in particular g-strain broadening, does not observably contribute to the linewidth.

Since the structure and orientation of the 12 neighboring Cu-Cu pairs are known from the crystal structure (Figure 3), the dipole–dipole contribution to the spectrum can be precisely calculated for 100% triplet population:

(5) $$H_{dip}=\sum_{i=1}^{12}\frac{\mu_{0}\beta^{2}}{4\pi r_{i}^{3}}\left[\left(\overset{̿}{g}\cdot \hat{S}\right)\cdot \left({\overset{̿}{g}}_{i}\cdot {\hat{S}}_{i}\right)-\frac{3\left({\hat{r}}_{i}\cdot \overset{̿}{g}\cdot \hat{S}\right)\left({\hat{r}}_{i}\cdot {\overset{̿}{g}}_{i}\cdot {\hat{S}}_{i}\right)}{r^{2}}\right]$$

in which $\hat{r}$ is a distance vector, and *r* is its absolute value.

A neighboring copper pair can, however, also be in the singlet, S = 0, state according to the Boltzmann distribution in Equation (2), and if that is the case, then there is no dipolar interaction with the central pair. With each of the 12 neighbors being either in the singlet or in the triplet state we end up with a total of 3896 configurations if each pair were uniquely discernible, and half of this number assuming inversion symmetry. To make the problem tractable, I assume an average interaction strength equal to m/12 times Equation (5) for a number of m neighbors in the triplet state, with a statistical distribution over state configurations F as:

(6) $$F={\left(1-N_{triplet}\right)}^{12}{N_{triplet}}^{m}\left(\frac{12!}{m!\left(12-m\right)!}\right)$$

**Figure 11:**
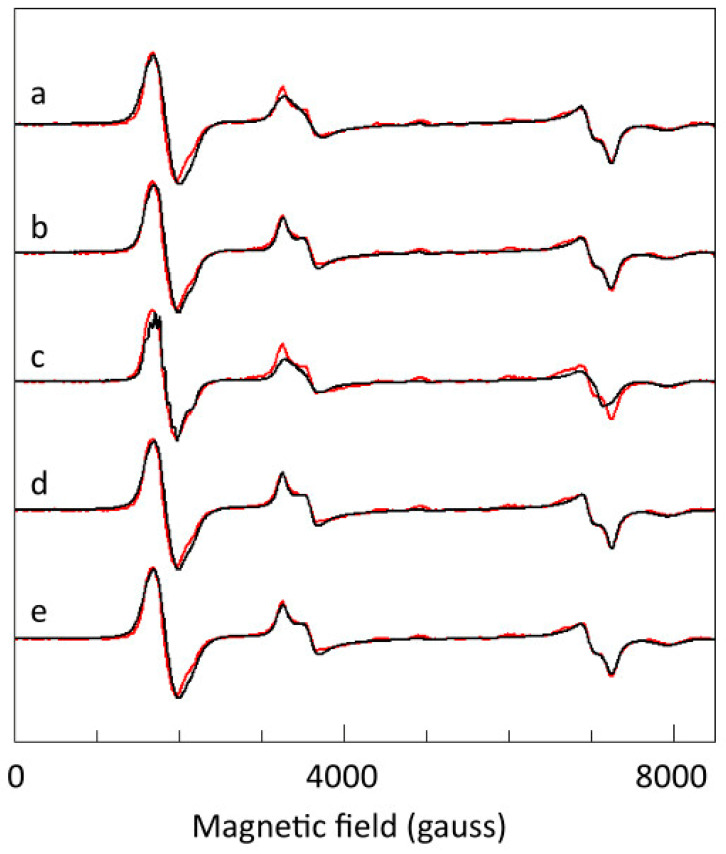
Exploration of contributions from different broadening mechanisms to the 150 K spectrum at 16.11 GHz. The simulations are as in Figure 10, top trace (where *W_zyx_* = 85, 85, 70 gauss) plus additional broadening, and with the Lorentzian linewidth from spin-lattice relaxation concomitantly reduced. In trace (**a**), *D* strain, or a Gaussian distribution in *D* values, is introduced with a standard deviation ∆*D* = 0.004 cm^−1^ around the average value of *D* = 0.335 cm^−1^ (∆*D* ≈ 1%), and *W_zyx_* = 65, 65, 50 gauss. This narrow distribution already results in a significant deterioration of the fit (notably, broadening of the X_3_Y_3_ feature), suggesting that *D* strain is insignificant. In trace (**b**), an *E* strain with ∆*E* = 0.004 cm^−1^ around *E* = 0.0105 cm^−1^ (∆*E* ≈ 38%), and *W_zyx_* = 75, 70, 53 gauss, does not significantly affect the fit. We have to increase, in trace (**c**), ∆*E* to 0.008 cm^−1^ (≈76%), and *W_zyx_* = 42, 42, 35 gauss, to notably deform the fit (broadening of the X_3_Y_3_ and Y_2_X_2_ features). In trace (**d**), dipolar interaction with 12 nearest-neighbor copper pairs is included, based on the point dipole model described in the text. No extra broadening is observed. However, if we assume, in trace (**e**), the dipoles to extend over the acetate bridges, which results in an effective reduction in the distance between pairs, then the simulation can accommodate the dipolar interactions when *W_xyz_* = 75, 85, 65 gauss. All in all, there is room for minor broadening by E strain and/or by dipolar interaction but only under a model of spatial dipoles. Simulations (**a**–**c**) were carried out with the program BB-TRIPLET; simulations (**d**,**e**) were made with the program DipolarTriplet. Frequency-independent contributions to the linewidth include broadening by a distribution in zero-field parameters, dipole–dipole interaction with neighboring copper pairs, and lifetime broadening. In Figure 11, it is shown that a distribution in *D* does not significantly contribute: a small standard deviation of ∆*D* = 0.004 cm^−1^ in a Gaussian distribution around *D* = 0.335 cm^−1^ deteriorates the fit with a specific broadening and amplitude reduction in the *Y*_3_*X*_3_ peaks compared to the rest of the spectrum. On the other hand, the parameter *E* can be distributed over a wide range (standard deviation up to ∆*E* = 0.0040 cm^−1^ with *E* = 0.0105 cm^−1^) without significantly affecting the overall shape of the simulation when the linewidth from lifetime broadening is concomitantly reduced (Figure 11). In other words, it is possible that *E* strain affords a minor but significant contribution to the linewidth, whereas lifetime broadening remains the dominant contributor.

To compute spectra resulting from such a distribution, Equations (5) and (6) were implemented in a simulation program, ‘DipolarTriplet’, described in Section 3, and listed in Supplementary Materials, using the spin Hamiltonian of Equation (1) extended with a hyperfine interaction for a symmetrical copper pair, and with the dipolar interaction of Equation (5). With the parameters of Table 1, and with the structure given in Figure 3, the calculation showed an insignificant contribution to inhomogeneous broadening at 150 K under the assumption of point dipoles centered at each copper pair (Figure 11). However, if the assumption is made that the dipoles have a volume that extends over the acetate bridges, that is, causing an effective reduction in length r in Equation (5), then a rather minor contribution of dipolar broadening to the overall linewidth is possible (Figure 11). 

Taking the above together, I conclude that the linewidth is dominated by frequency-independent lifetime broadening, Equation (4), possibly with unresolved contributions from a distribution in the rhombic zero-field parameter *E*, with a standard deviation up to maximally 0.004 cm^−1^, and from dipolar interactions between the copper pairs, but only when dipole moments extend onto the acetate bridges. All these broadening mechanisms are independent of the frequency, and the possible occurrence of the latter two cannot be experimentally distinguished from the case in which they would be completely absent. Each one of the spectra in the broadband collection of Figure 9 can now be individually simulated using a frequency-invariant linewidth. As a global analysis, this provides error bars to the parameters in Table 1.

### 2.5. Multi-Fequency Spectra Close to Zero Field

**Figure 12:**
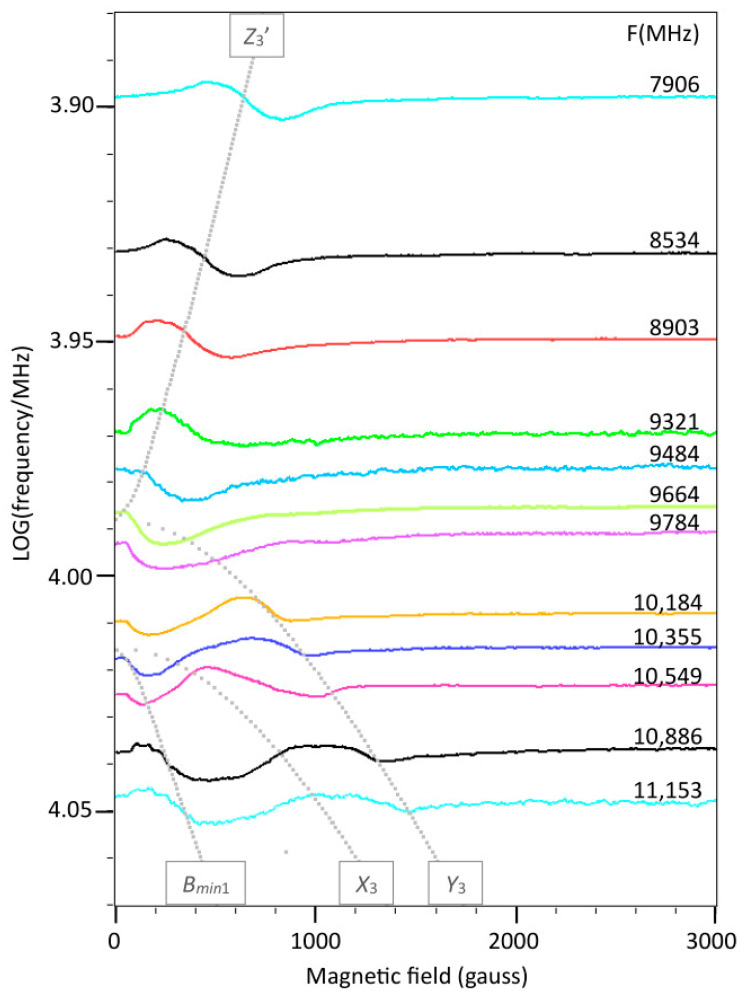
Zooming in on the zero-field transitions at *D* ± *E*. Spectra were recorded at closely spaced frequencies around the values 9728 and 10,358 MHz, which correspond to *hν* = *D* ± *E* cm^−1^ for *D* = 0.335 and *E* = 0.0105 cm^−1^. The dotted grey lines are canonical positions for resonances with finite amplitude calculated with the program Canonical Triplet Resonances. Note that at the zero-field frequencies the zero-field spectral shape sharpens to one half of a derivative feature in positive field. Since the electromagnet has a remnant field of circa 40 gauss, the first 40 gauss show a straight baseline with no spectral information. EPR conditions, microwave frequency as indicated; incident microwave power, 0–15 dBm; reflected power at the detection diode, 6–8.5 dBm; modulation frequency, 100 kHz; modulation amplitude, 25 gauss; temperature, 150 K.

Theoretically, in S = 1 spectra of systems with finite *D* and *E* values, specific resonances should disappear in zero field at three frequencies where hν corresponds to the absolute values of *D* + *E*, *D* − *E*, and 2*E*. In the wide frequency stack of Figure 9, it is difficult to see what exactly happens at zero magnetic field. Figure 12 zooms in on the *D* ± *E* area, at low magnetic fields, with 12 frequencies corresponding to a range of 2–3 frequencies in Figure 9. For |*D*| = 0.335 and |*E*| = 0.0105 cm^−1^ the zero-field frequencies are 10,358, 7728, and 630 MHz. The experiment of Figure 12 allows for some interesting observations. Starting from the high-frequency end, the Δm = 2 transition at *B_min_*_1_, with imperfectly resolved hyperfine splitting, smoothly moves into zero field around 10,358 MHz; however, the line never completely disappears: due to the finite linewidth, from lifetime broadening plus hyperfine interaction, there is always at least a negative lobe corresponding to half a peak in the integrated EPR absorption spectrum. Similarly, starting from the low-frequency end, the *Z*_3′_ resonance, with poorly resolved hyperfine splitting, moves into zero field around 7728 MHz, leaving half a peak detectable. The resonances *Y*_3_ (concomitant with *B_min_*_1_) and *X*_3_ (frequency-mirrored with *Z*_3′_) that also move into zero field, but with a steeper slope, appear to be the cause for the observation that in between the two zero-field frequencies the half line actually has a broader shape than exactly at the zero-field point. In summary, identification of the zero-field frequencies corresponding to *D* ± *E* by visual inspection requires searching for those two spectra where a half absorption line (that is, a derivative feature that starts with a negative slope) has its narrowest width. A minor technical complication is the blind spot that ensues from the circa 40 gauss remnant field of the electromagnet. Note that a determination of the zero-field frequency around 630 MHz for the 2*E* energy splitting is less reliable, because the resonances *Y*_2_ and *Y*_2′_ that move into and out of zero field have resonance field versus frequency slopes that are much less steep than the transitions around the *D* ± *E* frequency zeros (cf. Figure 9).

### 2.6. The Double-Quantum Line in Broadband EPR

In the classic treatment of Göppert-Mayer, a double-quantum absorption of radiation may occur in atomic spectroscopy when the sum of the energies of two quanta equals the energy difference between two atomic states:

(7) $$h\left(\nu_{1}+\nu_{2}\right)=\Delta E$$

provided that the intensities of the two waves are sufficiently high to overcome the lower transition probability compared to single-quantum transitions [42]. In continuous wave molecular EPR spectroscopy, this translates into *ν*_1_ ≡ *ν*_2_ since there is only one monochromatic microwave, and a high microwave power for a second-order process with the absorption amplitude, I, obeying [43]:

(8) $$I_{\mathrm{second}\;\mathrm{order}}\propto P_{W}^{3/2}\propto 10^{-{3P}_{dB}/20}$$

in which *B*_1_ is the field strength of the magnetic component of the microwave, *P_W_* is the microwave power in watt, and *P_dB_* is the microwave power in decibel attenuation from maximal input power. This contrasts with the power dependence of the first-order process of single-quantum transitions.

(9) $$I_{\mathrm{first}\;\mathrm{order}}\propto P_{W}^{1/2}\propto 10^{-P_{dB}/20}$$

Double-quantum EPR transitions that obey Equation (8) have been observed in the gas phase in O [44,45] and NO [46], in several organic triplet systems [47,48,49], and in transition ions Mn^2+^, S = 5/2, Ni^2+^, S = 1, and in Pr^3+^, S = 1, doped in cubic diamagnetic crystals [43,50,51,52,53,54,55,56].

A double-quantum transition of an apparently very different nature has been reported to be present in the EPR of a variety of Ni^2+^, S = 1, complexes of lower than cubic symmetry, usually studied in the form of pure, undiluted powders [7,22,24,57,58,59,60,61,62,63,64]. Its distinctive character first surfaced when two groups independently found its power dependence to follow Equation (9) for regular transitions, rather than Equation (8) for second-order processes [22,24]. A second distinctive property is the width of its absorption, which, in contrast to the very sharp double-quantum lines observed in doped cubic crystals, was found to be comparable, or only slightly reduced, compared to the linewidth of the regular triplet spectrum [22,24]. 

A third enigmatic feature was observed in some of the Ni^2+^ spectra, which exhibited a ‘negative’ double-quantum line, that is, the line had the shape of an emission rather than an absorption spectrum. This latter phenomenon was first seen in the EPR of a series of Ni(II) complexes with nitrogen donor ligands by Reedijk and Nieuwenhuijse, who chose to assign the negative peak to a double-quantum transition without further specification or explanation [57]. In fact, for one of the compounds, Ni(5-methylpyrazole)_6_(ClO_4_)_2_, the double-quantum line in my view appears to be positive in the X-band and negative in the Q-band (frequencies were not specified), and this remarkable fact was apparently unnoticed and left uncommented on [57]. Later, in a repeat recording of these spectra, Mabbs and Collison came to a different interpretation by assigning (the high-field half of) the transition in the X-band to be not a positive but a negative double-quantum line, although this resulted in a misfit of the assigned resonance field with the position in their spectral simulation [58]. In a subsequent study, Collison et al. made a full turn of opinion and decided that the ‘inverted phase’ line is not a double-quantum absorption, because an inverted line in the spectrum of Ni(II) doped in MgO crystal had previously been assigned to ‘internal cross relaxation’ [24]. For this latter statement the authors refer to early work by Sorokin et al., which, however, does not contain any data on inverted lines nor any discussion on relaxation [43]. Collison et al. furthermore reported that the Q-band spectrum is strongly temperature-dependent, with a continuous increase in the axial zero-field parameter *D* from 200 to 100 K, and with a step change around 200 K, below which temperature the double-quantum transition has a normal positive shape, whereas above 200 K an inverted line is found. Finally, in several of the spectra published by Collison et al. the double-quantum transition appears to have a more complex structure where a positive central derivative line has small side lines of opposite phase, which is not commented on by the authors [24]. All in all, the nature and properties of the first-order double-quantum feature in EPR are puzzling to say the least. More recent EPR work on Ni(II) complexes has not led to better insight. A half-field transition was erroneously named a double-quantum transition in [65]. A broad feature in a 611 GHz spectrum was assigned as a double-quantum transition, although the spin Hamiltonian parameters would predict the latter to be at a circa 10 kgauss lower field [7]. In high-frequency, low-temperature studies, double-quantum transitions were found with dispersion line shapes, which however received no comments [62,66].

**Figure 13:**
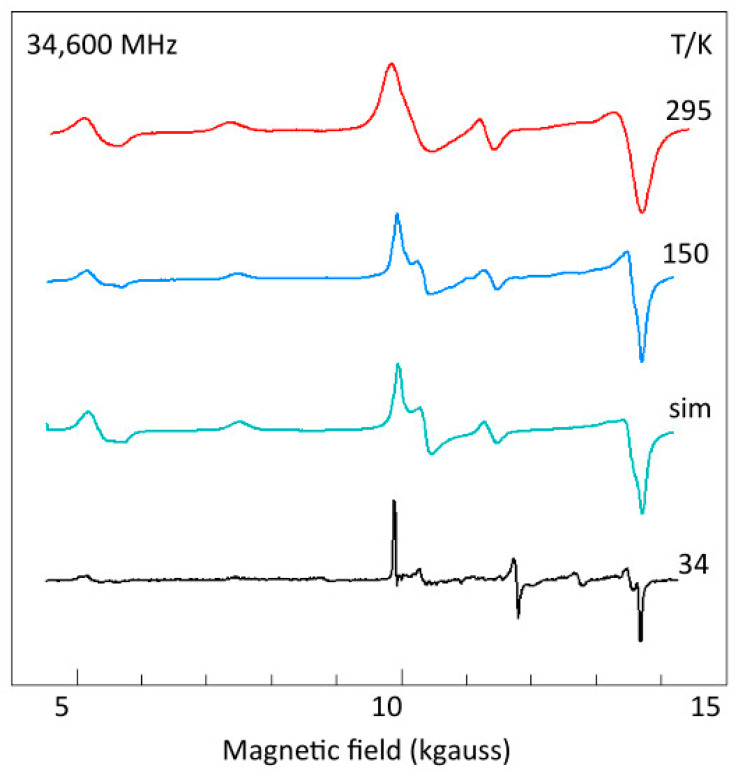
The Q-band spectrum of copper acetate monohydrate as a function of temperature. The spectra are complete: at this frequency, the *Z*_3_ transition overlaps with the *Y*_2_*X*_2_ feature. The third, green trace ‘sim’ is a simulation of the blue spectrum at 150 K based on the parameters in Table 1 determined at 16.11 GHz in Figure 10. As in the X-band (Figure 4), the Q-band spectrum shows increased resolution and decreased signal-to-noise ratio with decreasing temperature. Here, however, the double-quantum line is ‘right side up’, and its shape, width, and relative intensity are reproduced in the 150 K simulation without the need to introduce double-quantum transition-specific correction factors. EPR conditions: 34,600; 35,242; 34,750 MHz (the latter two re-calculated to 34,600 MHz); microwave power, −8 dB of 50 mW; modulation frequency, 100 kHz; modulation amplitude, 0.2; 5; 1 gauss; temperature as indicated.

All previous remarks on the first-order double-quantum line pertained to Ni(II) complexes. In Figure 4, I assigned a weak, apparently negative peak in the X-band spectrum of copper acetate monohydrate to a double-quantum transition. The peak can be recognized in earlier work on the complex [67] and on similar dimeric copper complexes [26,68], but it has not previously been assigned or otherwise commented on. A more noticeable, positive double-quantum peak I found at 16.1 GHz (Figure 10). Figure 13 gives Q-band spectra at 34.6 GHz as a function of temperature. The double-quantum line is now unmistakably present as a positive peak at higher temperatures. Just like in the X-band, at low temperature the linewidth has been reduced to the extent that overlap between the two transitions, |−1> → |0> and |0> → |+1>, which form the basis of the double-quantum transition at intermediate orientations, becomes negligible, and the line becomes undetectable. Proof for the assignment can be found in the simulation of the 150 K spectrum (Figure 13). Q-band spectra of copper acetate monohydrate have not been published before; however, the double-quantum transition can be seen in the literature containing spectra of similar copper-dimer compounds, initially unnoticed [69], then identified [27], then later labelled ‘*U*’, apparently for ‘unknown’ [70], and more recently assigned, without proof, to ‘interdimer exchange interaction’ [71].

**Figure 14:**
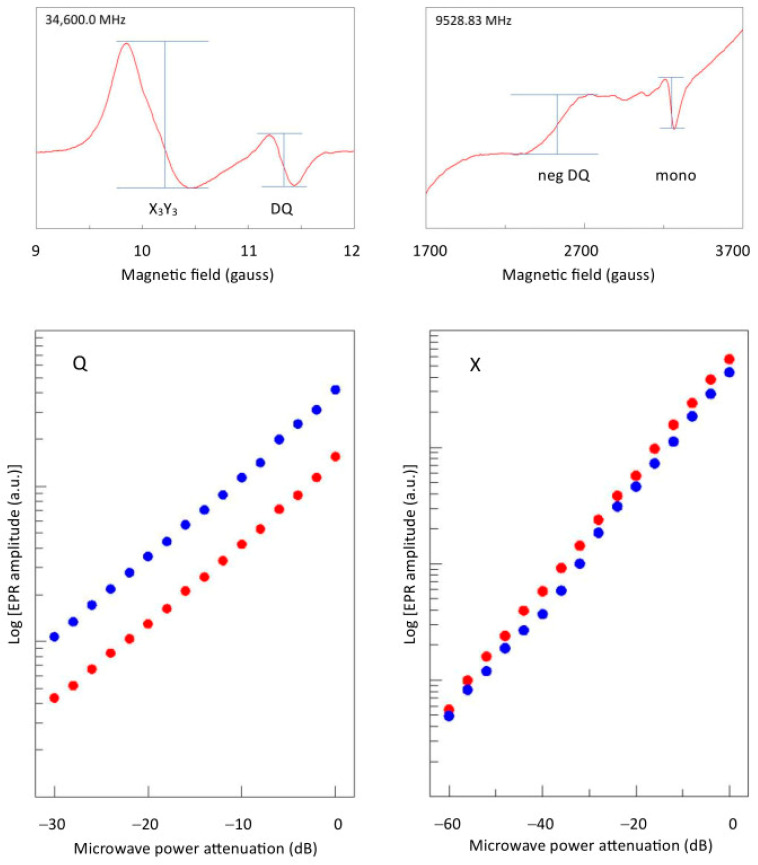
Power plots of the double-quantum transition in X- and Q-bands. The base-10 LOG of the intensity of the ‘right side up’ double-quantum transition in Q-band (left panel; red dots) at 295 K is plotted versus the attenuated power in dB of 50 mW, and compared to the intensity of the *X*_3_*Y*_3_ single-quantum transition (blue dots). Similarly, the intensity of the ‘upside down’ double-quantum transition in X-band (right panel; red dots) is plotted versus -dB of 200 mW and compared to the mononuclear Cu^2+^ signal (blue dots). Linearity of the data shows that the double-quantum transition is the manifestation of a linear process, that is, its amplitude is proportional to the power in dB, or to the square root of the power in mW.

Both the negative line in the X-band and the positive line in the Q-band exhibit the power dependence for a first-order process (Figure 14). For further analysis an imminent problem now is how to implement the generation of a first-order double-quantum signal in the simulation software. Collison et al. have proposed that first-order double-quantum transitions differ from second-order ones in the sense that the first are the result of a consecutive absorption of two quanta while the second represent the simultaneous absorption of two quanta. They hypothesize that the overall probability of the consecutive double-quantum transition, which they label ‘*P*’, should be the sum of the individual transitions: *P* = *P*_12_ + *P*_23_ (where the subscript indices 1–3 correspond to the levels |−1>, |0>, and |+1>). They also introduce two adjusting factors, namely, a pre-factor ≤ 1 for *P*_12_ to take care of possible relaxation from the intermediate level before transition to the top level can take place, and a linewidth for the double-quantum transition that is different from (and independent of) the linewidths of the regular spectrum [24]. The software in which these assumptions were implemented has not been published, and I suggest that we should not accept them at face value. A sum probability implies that a double-quantum transition could occur even if one of the composing transitions has zero intensity, which seems to be at odds with the definition of a double-quantum transition. Also, there does not seem to be any theoretical underpinning for a separate double-quantum linewidth. 

I have made the following alternative implementation of a double-quantum line in the BB-TRIPLET program. For every polar-angle step cos*θ* × *ϕ* on the unit sphere, that is for each molecular orientation, the shape, here Lorentzian, of the two transitions involved is calculated as for the regular spectrum. The two shapes are normalized to unit intensity, and then their product is constructed. This convolution is then multiplied with a linewidth-weighted absorption for the second transition. No adjusting factors are introduced. The main justification for this procedure is empirical: with reference to the Q-band spectrum at 150 K in Figure 13, the position, intensity, and width of the double-quantum transition are reproduced. Also, in my simulations (not shown) of published Ni(II) spectra [22,24], the algorithm reproduces the narrowing of the double-quantum line relative to the normal transitions in high-frequency spectra. On the other hand, in the fit to the 16.1 GHz spectrum in Figure 10 there appears to be some misfit where the experimental double-quantum line appears to be asymmetric with a decreased amplitude for the negative lobe on the high-field side. The phase inversion of the line in the X-band is not simulated at all.

**Figure 15:**
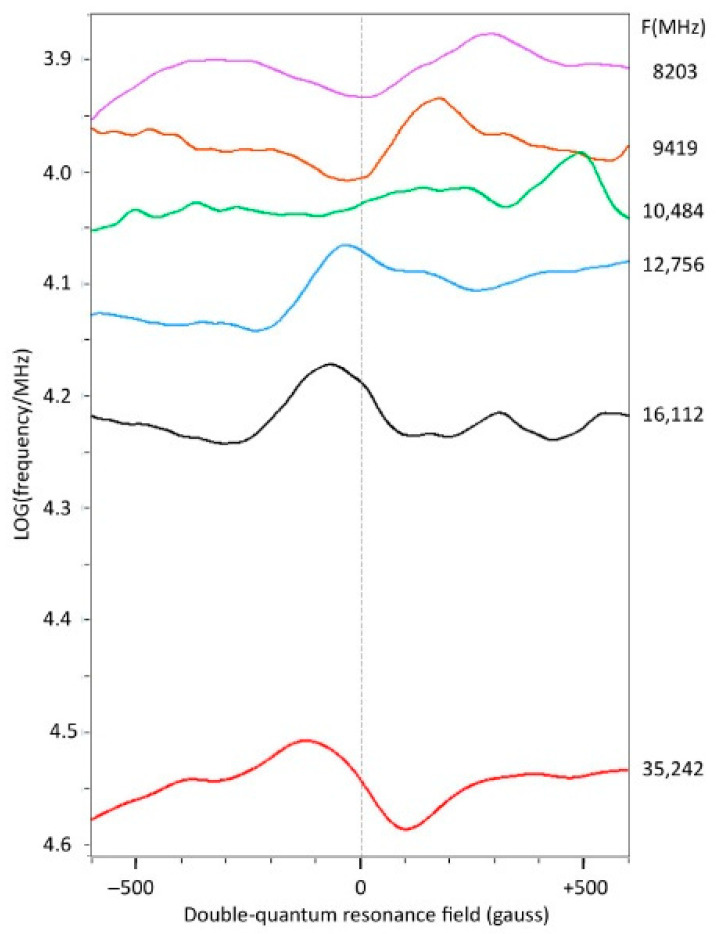
Stack of spectra from the double-quantum transition at higher frequencies. For each frequency, the field position of the double-quantum line in the powder spectrum was computed with BB-TRIPLET using the parameters in Table 1. Each spectrum was then centered with reference to this resonance field. The derivative-shaped line at 35.2 GHz gradually converts to a line with a dispersive shape at 8.2 GHz. EPR conditions as in Figure 9 and Figure 13 at 150 K.

In search of further insight into these matters, I have attempted to make a broadband analysis of the double-quantum line in copper acetate monohydrate where the spectra at different frequencies are aligned according to the field position of the zero crossing of the double-quantum absorption line calculated with the above algorithm. The stack is limited on the low-frequency site at 8.2 GHz, since at lower frequencies the double-quantum line becomes weak and eventually confluences with the regular *Y*_2_*X*_2_ feature. Figure 15 shows that at 35.2 GHz the line has a regular shape, but at 16.1 GHz, and more so at 12.8 GHz, it progressively deforms asymmetrically, until it becomes virtually undetectable at 10.5 GHz. At 9.4 GHz, the line reappears at first sight as an inverted peak, but at closer inspection it turns out that it is actually the negative lobe of the inverted line that coincides with the calculated double-quantum resonance field. This behavior continues at 8.2 GHz where the overall shape now has the approximate appearance of a dispersion line rather than an inverted absorption line. Furthermore, the line in the Q-band is flanked by minor features, which perhaps are also present at the other frequencies with deformation. This result, combined with the repeated documentation of apparently inverted double-quantum lines in Ni(II) spectra at some frequencies but not always at others, plus the satellite lines that I observe in some published Ni(II) spectra, lead me to conclude that first-order double-quantum lines in spectra of transition ion complexes are manifestly much more complex than hitherto assumed, and are worthy of future experimental and theoretical studies.

### 2.7. The Copper Hyperfine Interaction

With two copper ions, each with a nuclear spin I = 3/2, the spin Hamiltonian in Equation (1) is extended with a term:

(10) $$H_{hyp}=S\cdot (A_{1}\cdot I_{1}+A_{2}\cdot I_{2})$$

in which a small difference in nuclear magnetic moment between the two stable copper isotopes is ignored. With full equivalence between the metal ions, a hyperfine pattern is expected of two overlapping quartets resulting in a seven-line pattern with relative intensities 1:2:3:4:3:2:1. This motif was indeed observed in the seminal single-crystal EPR study of Bleaney and Bowers on copper acetate monohydrate, in which they estimated the components of a colinear hyperfine tensor to be *A_z_* = 70 gauss and *A_xy_* < 9 gauss [25]. In a subsequent study on single crystals doped with Zn(II), Kokoszka et al. determined the hyperfine tensor components of the monomeric copper complex as *A_z_* = 157 gauss and *A_xy_* < 19–25 gauss [41]. These values should be divided by two to obtain the equivalent numbers for the dimeric complex. Hyperfine values for copper acetate monohydrate in powder form have never been reported, but in the spectra of other dimeric copper complexes spectral simulations afforded *A_z_* values of 72–79 gauss [26,71] and *A_xy_* unresolved.

**Figure 16:**
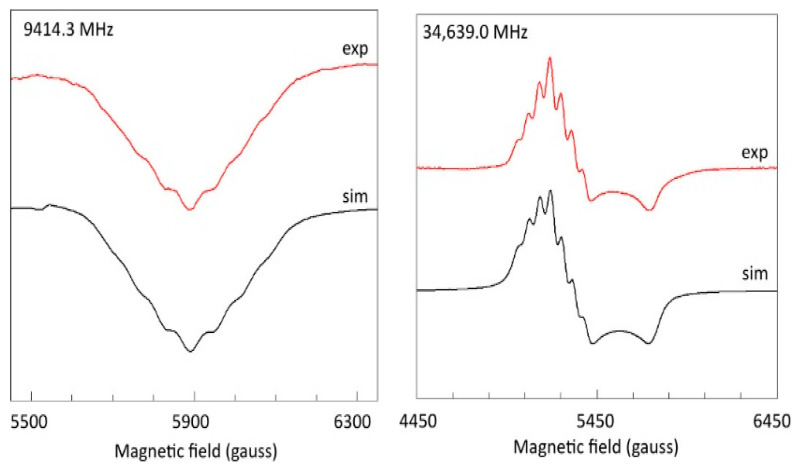
Copper pair hyperfine structure in X- and Q-band spectra of copper acetate monohydrate. The first-order hyperfine pattern with intensity ratio 1:2:3:4:3:2:1 was simulated in X-band (left panel) with parameters *g_z_* = 2.361; *A_z_* = 64 gauss; *W_z_* = 40 gauss; and the zero-field parameters of Table 1. EPR conditions: microwave power −10 dB of 200 mW; modulation amplitude, 1 gauss; temperature, 136 K. In Q-band (right panel) the simulation used *g_zyx_* = 2.365, 2.04, 2.07; *A_zyx_* = 64, 1, 1 gauss; *W_zyx_* = 40, 40, 40 gauss; and the zero-field parameters of Table 1. EPR conditions: microwave power −8 dB of 50 mW; modulation amplitude, 5 gauss; temperature, 110 K.

In the *Z*_3_ peak in the X-band at 150 K, a poorly resolved hyperfine pattern is found (Figure 16, left panel) very similar to the pattern originally detected in single crystals [25]. However, the simulated splitting *A_z_* ≈ 64 gauss is significantly smaller than reported for the single-crystal spectrum. In the Q-band (Figure 16, right panel), the Δ*m_S_* = 2 feature affords a better resolution, where a simulation equally results in *A_z_* = 64 gauss. This value has been used in all simulations throughout this study. As in all previous studies, *A_x_* and *A_y_* remain unresolved, and in all simulations a dummy value of *A_xy_* = 1 gauss has been used.

## 3. Materials and Methods

The triplet model compound [Cu(II)(acetate)_2_H_2_O]_2_, 99.99% pure based on trace metal content, was obtained from Sigma-Aldrich, and was ground in a mortar to a fine powder of ≤0.1 μm particle diameter. The fractional coordinates (including protons) in the monoclinic unit cell from neutron-diffraction analysis [33] were converted to Cartesian coordinates, from which a file was constructed in pdf format to generate the structure in Figure 3A. Repeated unit translation of the fractional coordinates along the crystal axes provided a data set that, after conversion to Cartesian coordinates, was the basis for the nearest-neighbor crystal pictures in Figure 3B,C.

Single-frequency data were collected with conventional spectrometers operating in the X-band (Bruker EMX-plus with standard ER4102ST rectangular resonator; maximum output power 0 dB = 200 mW) and in the Q-band (Varian E-line with cylindrical resonator; maximum output power 0 dB = 50 mW). Home-build helium-flow cryogenics were as in [72,73]. Multi-frequency data were obtained with the most recent version of the broadband EPR spectrometer, consisting of the conventional Bruker EMX-plus spectrometer extended with a multi-frequency conversion kit to replace the X-band bridge [13]. The source power is expressed in absolute units of dBm, where 0 dBm ≡ 1 mW. The setup uses home-made multi-frequency cells based on wire microstrip technology to allow for tuning to many frequencies in the approximate range of 0.1–18 GHz [10,13]. The modulation-coil assembly was taken from the Varian E-line Q-band spectrometer, and was combined with the original Q-band flow Dewar [73]. The sharp isotropic EPR line (g = 2.00254) from BDPA (α,γ-bisdiphenylene-β-phenylallyl) complex with benzene (1:1), purchased from Sigma-Aldrich, was used for calibration of the magnetic field. The single-line EPR spectrum (g = 2.0036) of DPPH (2,2-diphenyl-1-picrylhydrazyl) from Sigma-Aldrich was used to calibrate the modulation amplitude and to optimize the signal phase in the signal-channel unit of the EMX-plus spectrometer both in X-band and in multi-frequency setup. 

Software was written in LabVIEW 2020 to construct convenient user interfaces, with calls to dynamic link libraries (DLL) written in Intel-FORTRAN for the computationally intensive sub-procedures, using the Intel Visual Fortran Compiler 2020 integrated into the Microsoft Visual Studio Community 2019 development environment. Software that was written for the present work is cited in the text. Executables and complete source-code listings can be found in Supporting Information. Specifically, the program ‘BB-TRIPLET’ generates 1024-point S = 1 spectra for perpendicular-mode EPR for a given set of *g_xyz_* and *D*, *E* values. The latter may be chosen to be statistically varied according to a Gaussian distribution. The program includes a first-order hyperfine interaction for a dimer of equivalent copper ions. The line shape can be Gaussian or Lorentzian, and linewidth parameters may be defined in gauss or in *g*-value units (*g* strain). A first-order double-quantum transition sampling the seven hyperfine lines is included as described in Section 2.6. The program also reports the resonance field (zero crossing) for the double-quantum line for the construction of plots like Figure 16. For direct comparison with experimental spectra, the program reads ascii files of 1024 amplitude values headed by 10 comment lines, where the third line should give the experimental frequency (GHz), start field (gauss), and end field (gauss), separated by a comma and a blank space. The program ‘CanonicalTripletResonances’ traces resonance fields and transition probabilities as a function of LOG(frequencies) for all spectral turning points at the molecular axes plus the *B_min_*_1_ turning point at intermediate orientation. It generated the grey lines in Figure 9 and Figure 12.

In its present form, the program Dipolar Triplet is specific to copper acetate monohydrate. It generates EPR spectra broadened by dipole–dipole interaction with 12 nearest-neighbor copper pairs (cf. Figure 3B,C). Triplet-state population is corrected for temperature. Broadening is averaged over the 12 neighbors according to Equations (5) and (6). Interaction is either based on the point-dipole model between copper pairs or on the extended dipoles model between closest points on acetate bridges. Double-quantum transitions are not calculated in this program, nor are second transitions from crossing energy levels.

## 4. Conclusions

1. Low-field broadband EPR spectroscopy allows for comprehensive study of S = 1 systems, in particular when the axial zero-field splitting parameter D is comparable to the X-band quantum or less;
2. All regimes *D* > *hν*, *D* ≈ *hν*, and *D* < *hν* can be covered;
3. All three zero-field transitions at *hν* = *2E*, *D* − *E*, and *D* + *E*, can be sampled;
4. Global analysis of broadband S = 1 EPR gives spin Hamiltonian parameters with error estimates;
5. The EPR of copper acetate monohydrate is rhombic (*g_x_* ≠ *g_y_*; *E* ≠ 0) consistent with deformation by hydrogen bonds in a plane;
6. Broadband EPR allows for unequivocal line shape and linewidth analysis, e.g., in the present case of copper acetate monohydrate a homogeneous Lorentzian line shape from T_1_ relaxation is found with no, or negligible, inhomogeneous contributions from *g* strain, *D* strain, and/or unresolved superhyperfine splittings, but possibly with finite contributions from *E* strain;
7. Contributions to the linewidth from dipolar interactions are negligible under the assumption of the point-dipole model, however, they may be significant under a spatial dipole model;
8. Spin-lattice relaxation includes a thus far rarely encountered inverted Orbach mechanism, which–contrary to regular Orbach relaxation–is virtually temperature-independent;
9. At temperatures below 150 K, the *X*_2_ resonance continues to sharpen but the *Y*_2_ resonance re-broadens; the latter phenomenon is not understood;
10. Broadband EPR of copper acetate monohydrate encompasses a linear double-quantum transition, with a complex line shape dependency on frequency, which calls for future experimental and theoretical studies.

## Figures and Tables

**Table 1:** Spin Hamiltonian parameters from simulation.

|*D*| = 0.335 (0.002) cm^−1^	|*E*| = 0.0105 (0.0003) cm^−1^	*E*/*D* < 0
*g_z_* = 2.365 (0.008)	*g_y_* = 2.055 (0.010)	*g_x_* = 2.077 (0.005)
*A_z_* = 64 gauss	*A_y_* = 1 gauss	*A_x_* = 1 gauss (dummy value)
*W_z_* = 85 gauss	*W_y_* = 85 gauss	*W_x_* = 70 gauss

Notes: initial simulation values were taken from [30,41]; data were fitted at 16,112 and 12,756 MHz (with identical results) since only at these frequencies does the spectrum show all the resonances as defined in Figure 2. Data at lower frequencies down to 2930 MHz were fitted to determine standard deviations of spin Hamiltonian parameters.

## Data Availability

The data are contained within the article.

## Supplementary Materials

The following supporting information can be downloaded at: https://www.mdpi.com/article/10.3390/ijms241914793/s1.
